# Circadian hormone secretion of enteroendocrine cells: implication on pregnancy status

**DOI:** 10.3389/fendo.2023.1106382

**Published:** 2023-05-10

**Authors:** Abdelgadir M. Homeida, Mohamed A. Homeida, Ebtesam A. Al-Suhaimi

**Affiliations:** ^1^ Department of Environmental Health Research, Institute of Research and Medical Consultations Imam Abdulrahman Bin Faisal University, Dammam, Saudi Arabia; ^2^ UH Cleveland Medical Center, Case Western Reserve University, Cleveland, OH, United States; ^3^ Department of Biology, College of Science, Imam Abdulrahman Bin Faisal University, Dammam, Saudi Arabia

**Keywords:** enteroendocrine cells, circadian rhythm, hormone, intestine, pregnancy, human

## Abstract

The timing of food intake is a key cue for circadian rhythms in humans and animals. In response to food intake, gut hormones called incretin are produced by intestinal enteroendocrine cells in a circadian rhythm that stimulates insulin secretion and regulates body weight and energy expenditure. Pregnancy is associated with the expansion of β cells, the risk of gestational diabetes mellitus, and excessive weight gain. The timing of food intake is a good way to address metabolic complications during pregnancy. The current review focuses on the circadian rhythms and biological actions of enteroendocrine hormones and their associations with pregnancy status, specifically topics like food intake and gut circadian rhythms, the circadian secretion of enteroendocrine peptides, and the effects of these factors during pregnancy.

## Introduction

Endogenously developed rhythms that take place over a period of almost 24 h are known as circadian rhythms. These rhythms play a vital role in the survival and progression of life. Everyday rhythms of physiology and behavior are managed by the circadian rhythm system, which allows creatures to forecast recurring alterations in the surrounding environment and establish critical acclimatized systems. Circadian rhythms also allow for the optimal use and production of energy (1). The mapping of nearly entire sides of physiological functions (body temperature, hormone secretions, sleep–wake cycles, etc.) occurs across these 24-h rhythms. Nonetheless, circadian rhythms are often interrupted by contemporary lifestyles. These variations in circadian rhythms are found to be significant contributory factors (2).

The central pacemaker of the circadian rhythm in mammals lies in the hypothalamus, known as the suprachiasmatic nucleus (SCN). SCN plays a crucial role in the maintenance of systemic circadian rhythms and regulates peripheral tissue clocks through the secretion of endogenous regulatory factors (3). The molecular clock of the circadian system, which is present in all cells, is made up of oscillating clock-related proteins that compose transcription and translation feedback loops (TTFLs) (4). The core TTFL is composed of the transcriptional activator proteins CLOCK and BMAL1 and the repressor proteins Period-1 (PER1), Period-2 (PER2), Period-3, Cryptochrome-1, and Cryptochrome-2 (4). Other loops are coupled with the core TTFL to maintain oscillation.

**Figure 1:**
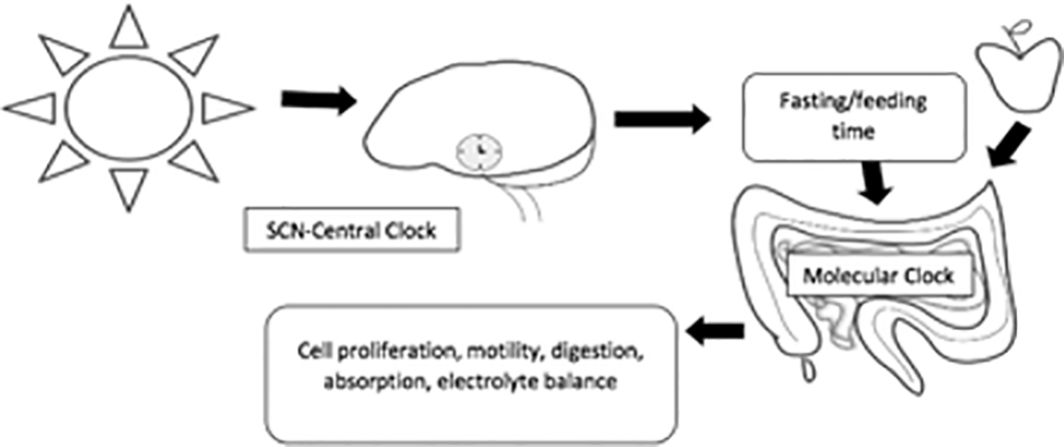
The main feedback loops regulating the circadian clock. In mammals, all tissues possess circadian oscillators, making the system organization highly complex. The light-entrainable pacemaker is in the suprachiasmatic nucleus (SCN) of the hypothalamus, and its function is to synchronize peripherical molecular clocks. While all these oscillate within a period close to 24 h, it is essential that they are synchronized with the external environmental conditions. Hence, the key function of the SCN clock is to receive environmental light information from the retinohypothalamic track and synchronize other molecular oscillators, both within the SCN and in peripheral organs.

The circadian rhythm is mainly entrained by environmental signals, such as light, food, and arousal stimuli. In the SCN, the circadian clock mainly responds to the light–dark cycle (**Figure 1**). In peripheral tissues, the circadian rhythm can be synchronized by food or temperature (4). Moreover, internal signals, such as circulating hormones, metabolites, sympathetic nervous activation, and body temperature, are significant timing cues that regulate peripheral clocks (3).

The SCN is in conformance with the day–night cycles through the retina, which stimulates the pineal gland to produce the melatonin hormone (1) and also regulates the rest of the oscillators throughout the body systems to target melatonin’s receptors in different organs. A daily rhythm is adopted by the plasma melatonin, where there are high levels at night. Hence, it is known as the hormone of darkness (5, 6). The vital source of the hormones serotonin and melatonin external to the CNS is the gastrointestinal tract. A vital role is performed by these two chemicals in gastrointestinal motility (2).

Melatonin release rhythm is one of the outputs of the SCN but not the exclusive one. The C57BL6 mouse strains are deficient in melatonin synthesis due to mutations in genes coding two key enzymes of the melatonin synthesis pathway, namely, arylalkylamine-*N*-acetyltransferase and *N*-acetylserotonin-*O*-methyltransferase, while they have functional autonomous circadian rhythms under constant conditions (7). Inbred strains of mice (*Mus musculus*) kept under LD 12:12 cycles were used to study pineal gland melatonin levels (8). The results have indicated that only five inbred strains have pineal melatonin content, with higher levels during the night and lower levels during the day; the other 31 strains do not contain detectable melatonin in their pineal gland in any of the times examined. The former group includes two commonly used strains (C3H/He and CBA/Ms) and three wild-derived strains (Mol-A, Mol-Nis, MOM). C3H and CBA mice showed a similar pattern of pineal melatonin rhythm with a peak at 2 h before lights on (8, 9). These results confirm that melatonin plays a supportive instead of an essential role in maintaining circadian rhythms in mammals.

In addition to melatonin, one pathway by which the SCN is known to influence downstream processes is through the regulation of sympathetic tones (10, 11). Treatment with 6-hydroxydopamine, a neurotoxin that destroys sympathetic terminals and reduces the amplitude or abolished circadian patterns for stool number and weight, suggests that sympathetic activity is required for sustained circadian patterns of intestinal motility (12). Administration of the β-adrenergic agonist isoproterenol causes a phase-dependent shift in PER2 expression rhythms. Collectively, the data suggest that the SCN is required to maintain feeding, locomotor, and stool output rhythms during *ad libitum* conditions, acting at least in part through the daily activation of sympathetic activity (12).

Glucocorticoids may also mediate the actions of SCN in the intestine. The best-characterized regulation of the circadian release of glucocorticoids is *via* the hypothalamic–pituitary–adrenal axis controlled by the SCN (13). The extra-adrenal production of glucocorticoids may also occur in the intestine (14). In the gut, for example, steroidogenic enzymes, such as StAR, CYP11A1, and 3βHSD, have been detected in mouse gut epithelium, which can produce glucocorticoids *de novo* (14). Similarly, CYP11A1 and CYP11B2 are present in human colon biopsies, and human colonic tissue can produce cortisol when cultured (15). The possible functions of glucocorticoids may include the regulation of local immune homeostasis and epithelial barrier integrity (16), maturation and differentiation of epithelial cells (17), and expression of tight junction proteins (18).

Orexin and melatonin have been studied as central neuroendocrine transducers, while leptin, ghrelin, and cortisol are three peripheral hormones required in the circadian system for synchronization in mammals. They act as key internal transited messengers and inputs for other endogenous oscillators (16–18). Therefore, the exploitation of orexin receptor agonists and antagonists, such as almorexants, is helpful for narcolepsy and extensive daytime sleepiness (16).

The circadian system is formed by a network of oscillators found in central and peripheral tissues that are tightly linked to generate rhythms in vertebrates to adapt the organism to cyclic environmental changes. The nuclear receptors PPARs, REV-ERBs, and RORs are transcription factors controlled by the circadian system that regulate, among others, a large number of genes that control metabolic processes for which they have been proposed as key genes that link metabolism and temporal homeostasis (19).

Enteroendocrine cells produce over 12 different hormones reliable for some processes, such as gut motility, digestion, food absorption, metabolism, and coordination (1). The current review focuses on the hormones that exhibit circadian rhythms and their biological actions and association with pregnancy status.

The intestine hormone-producing enteroendocrine cells are distributed lengthwise along the epithelium layer through the gastrointestinal (GI) tract (1). The gut hormones regulate secretory and motility functions in the GI tract. Furthermore, they control appetite and energy expenditure, mainly *via* the gut–brain axis, as well as glucose homeostasis through effects on pancreatic hormone secretion (2, 20). Knowledge of the physiology of intestine hormones reveals the advancement of two categories of antidiabetes treatments, such as dipeptidyl peptidase-4 inhibitors, a drug that was approved for obesity. Another drug is glucagon-like peptide-1 (GLP-1) mimetics (21, 22).

## Gut functions and circadian rhythms

In addition to central clocks, peripheral biological clocks are present in the majority of tissues, if not all of them, such as the gut, liver, retina, and heart (23). Robust circadian oscillations are demonstrated by the explants and cultured cells extracted from those tissues in gene expression (24). The expression of these clock genes and their rhythmic regulation are not unique to the SCN but instead are widely distributed in many cells and tissues. For instance, the *period* genes are expressed and rhythmically regulated in a variety of peripheral tissues including the liver, lung, and skeletal muscle (25). Various gut functions are controlled by the molecular clock, either dependent or independent of the SCN. These include endobiotic and xenobiotic detoxification, nutrient absorption, and motility of large intestines (26).

Different biological rhythms are also shown by the gut. Vital core activities of the gut are influenced by diurnal oscillations, such as maintaining and changing the protective barrier, motility, secretions, and gut microbiota (27).

Circadian rhythm stability is pivotal for the maintenance of mucosal barrier function. Circadian rhythm disruption increases intestinal necroptosis, thus rendering the gut epithelium more susceptible to inflammatory processes (28). It has been found that disturbances in circadian oscillators may directly and indirectly (through melatonin and other circulatory elements) affect protecting barriers in the mucosal layer of the GI and cell multiplication (29). A potential relationship between shifting the job and development of duodenal ulcers was recently reported by Pietroiusti et al. (27). It was asserted by the authors that in comparison with daytime workers, there was a higher incidence of duodenal ulcers in shift workers (27). Nonetheless, there continues to be a weak understanding of the mechanisms responsible for this occurrence. A reduction in circulating melatonin because of the shift work in the night may be a contributing factor to this phenomenon. Melatonin application accelerates gastric ulcer healing and can physiologically regulate antioxidative enzyme activity and increase gastric blood flow level (29).

There is a marked circadian variation in gastric luminal human trefoil protein (TFF2) in young healthy volunteers with peak levels present during the night. It is demonstrated that the TFF2 rhythm is impaired in cohorts of individuals known to suffer gastric symptoms of *Helicobacter pylori* infection (30). In the elderly, the amplitude of the circadian rhythm of TFF2 is considerably decreased, which may influence the protective systems of the GI mucosa (30, 31).

Secretory changes in the gut are also influenced by circadian rhythms, particularly modifications to HCl release (32, 33). Gastroesophageal reflux disease (GERD) is a common illness related to gastric hypersecretion. GERD patients often make complaints about recurring reflux symptoms at night. Their quality of life is adversely affected by night-time GERD, as it creates pain and disrupts sleep, which has an impact on the mental and physical activity of the person the following day. Furthermore, there are certain GERD patients who are taking antisecretory medicines, such as PPIs, and for them, a description of the nocturnal acid breakthrough phenomenon has been provided (33, 34).

Clinical investigations have been provided as support for the significance of the relationship between the molecular clock and colonic motility in clinical investigations that depict a widespread incidence of functional colonic motility disorder because of the interference in circadian rhythms (34). The relationship between alternative jobs and the existence of efficient bowel dysfunction was examined by Nojkov et al. (33). This study comprised 399 nurses that were involved in patient care. The groups examined included 214 of them during the day period, 110 nurses from the night period, and 75 who practiced alternating shifts. It was found that alternating shifts can have a substantial effect on the efficient illness of the bowels.

The aging process has one of the most powerful impacts on the circadian mechanism. To sum up, it was noticed that the following changes occurred in the circadian system: 1) age-linked modifications in the central biological clock, such as a reduced expression of AVP and VIP neuropeptides that led to a decline in circadian electrical activity amplitude and lessened sensitivity to the melatonin hormone in the SCN; 2) age-linked modifications in diurnal entrainment (a reduced response to the stimulus of light and nutrition entrainment and a decrease in melatonin concentrations in circulation); 3) changes in the genetic factor clock because of aging (lower expression of significant genes in both the master clock and peripheral tissue); and 4) incidences of efficient GI illnesses because of aging and the neurodegenerative effect of cholinergic degeneration (35–37).

Bacteroidetes and Firmicutes dominate the gut microbiota. Nonetheless, there are considerable interindividual variations in microbial composition (38). The source of this variation is the integrated impact of host genetics, physical location, aging, diet, lifestyle (39), and pet ownership (40). Confusion prevails regarding most of these factors.

It is important to have a balanced gut microbial constitution in host physiology, and it is believed that compositional disturbance leads to several illnesses. For example, it is believed that obesity causes a considerable decrease in Bacteroidetes and a subsequent elevation in Firmicutes (41).

Circadian rhythms exist in prokaryotes as well as in Cyanobacteria (42). Diurnal sequences of light and warmth are faced by free-living bacteria; hence, their circadian rhythm permits them to forecast and adapt to variations in ecological statuses.

The time-of-day-based constitution of the rat fecal microbiota was reported by Thaiss et al. (43). Routine oscillation is experienced by over 15% of identified operational taxonomical units (OTUs) in their comparative richness. These include many species, such as *Lactobacillus reuteri* and *Dehalobacterium* spp., which are part of Clostridiales, Lactobacillales, and Bacteroidales. Later on, two more groups offered further support. According to Zarrinpar et al. (44), there is a cyclic nature in 17% of OTUs. These everyday modifications are evident in the variety and constitution of the GI microbiota (45). There is a diurnal variation in the gastrointestinal ecosystem, which is subject to the time of the day and the status of food and fasting. However, the host circadian rhythms are also influenced by the microbiota (46). If the diet is adjusted, the constitution of the gut microbiome can rapidly change, which may cause the circadian rhythmicity to be modified (47). Night-shift work and induced chronodisruption have been linked in various studies to colorectal and stomach cancer (48). It is suggested that colorectal cancer can be developed as a result of the changes in the microbiota following a disruption to circadian rhythms. An irregular composition of the microbiota environment in mice was related to inflammation and tumors (49).

## Circadian rhythm and enteric endocrine hormones: glucose-dependent insulinotropic polypeptide

Enteroendocrine K cells situated in the duodenum and proximal jejunum secrete glucose-dependent insulinotropic polypeptide (GIP), which is a gastric inhibitory hormone made up of 42 amino acids. The incretin effect is the process of a glucose-dependent insulin release that is regulated by GIP and glucagon-like peptides. GIP plays a significant role as an incretin hormone that regulates the levels of blood glucose. GIP and GLP-1 are secreted as endocrine hormones in the blood after eating (**Table 1**) and are responsible for up to 70% of insulin release as a response to meal intake (50–52). Diurnal fluctuations are shown (**Table 1**) following the secretion of GLP-1 right before their active period, and increased responses are shown at ZT16 in rats (52).

**Table 1:** Circadian variations of some incretin hormones after 30 min of ingestion of standardized mixed meal in healthy men or after 10 min of oral glucose load in rats.

Parameter (mean ± SEM), humans	Morning (08:00 a.m.)	Evening (07:00 p.m.)	Reference
GLP-1 (pmol/L)	35.00 ± 3.00	28.00 ± 2.00	(50)
GLP-1 (pmol/L)	1.28 ± 0.25	0.90 ± 0.06	(51)
GIP (pmol/L)	90.00 ± 4.00	65.00 ± 4.00	(50)
GIP (pmol/L)	126.00 ± 6.00	95.00 ± 4.00	(51)
Parameter, rats	ZT5 (light)	ZT16 (dark)	
GLP-1 (pg/ml)	7.90 ± 0.90	22.50 ± 3.10	(52)

A diurnal pattern is also shown by insulin secretion, with increased secretion taking place in terms of the active/feeding period in humans and rodents (53). In addition, it has been demonstrated in earlier studies that routine insulin-releasing rhythms exhibit a more distinct response to oral feeding than IV feeding, which shows that incretin hormones play a vital role in promoting circadian insulin release (53). The stimulation of GIP receptors (GIPR) on pancreatic β cells is found to regulate GIP (54). In addition, it has been shown in other studies that GIPR signals exhibit favorable effects that support mineral deposition in bones (55).

It is interesting to note that when the eating–fasting plan is modified for the same experimental animals, a parallel shift in peak GIP secretion occurs (54), which indicates that the major zeitgeber for the diurnal rhythm in GIP is the intake of nutrients. Variations in the pattern of GIP circadian secretion in diabetic, obese, or lower body-weight individuals indicate that the release of hormones may be affected by metabolic status (40).

A specific effect of GIP to stimulate the secretion of intestinal GLP-1 was demonstrated *in vivo* in rats (55). This enteroendocrine loop between the duodenal peptide GIP and the ileal GLP-1 may account for some of the early rises in the secretion of GLP-1 observed in response to nutrient ingestion.

## Glucagon-like peptide-1

GLPs are released by enteroendocrine L cells found in the distal small part and large intestine (54). It is the enteroendocrine L cells that create this insulinotropic peptide. GLP-1 is an incretin hormone, and similar to GIP, it is secreted soon after a carbohydrate meal and taken to support insulin secretion, which also supports the release of glucose-dependent insulin. Food intake, stomach emptying, and glucagon release are restricted by GLP-1, which brings about β-cell differentiation, propagation, and cell neogenesis. The expression of the GLP-1 receptors occurs in different metabolically active tissues, which triggers various biological effects throughout different organ systems (54).

Research on humans indicates that GLP-1 release is temporally regulated, showing that GLP-1 has higher efficiency during the early hours of the day in comparison with the late hours of the day for participants that are administered similar meal programs with varying periods of fasting (50). A distinct release of GLP-1, depending on the time of the day, was decreased when subjects came across nocturnal light (56). Phase-delayed circadian misalignment increased rapid eye movement sleep and sleeping metabolic rate, increased glucose and decreased GLP-1 concentrations, and increased carbohydrate oxidation that may create a health risk through a metabolic disturbance (56). It has now been determined with carefully regulated studies of rats and mice that a GLP-1 release adopts a substantial 24-h secretory manner in reaction to similar levels of glucose that are given by following a similar duration of fasting (52, 57). In rodents, it was determined that the peak level of GLP-1 release was at the start of the dark/eating phase, while the lowest release occurred at the start of the light/fasting phase (52, 57), which means that the key zeitgeber of the L cell is nutrient intake, demonstrating the existence of a diurnal rhythm in GLP-1 secretory responses to an oral glucose load in rats, with increased release immediately preceding the normal feeding period. This profile of GLP-1 release correlated with the pattern in insulin secretion, and both rhythms were completely inverted in animals subjected to a 12-h feeding cycle disruption and abolished in rats maintained under constant light conditions (52). Interestingly, the intestinal microbiome was established to be an integral component of the pathway regulating diurnal GLP-1 release (57).

It was demonstrated that the core SNARE protein syntaxin-1a (syn1a), which is expressed by murine ileal L cells, plays an essential role in secretagogue-induced exocytosis of GLP-1 (58). Munc18-1 (Syntaxin binding protein-1), a regulator of membrane fusion, further controls vesicle docking and secretion by interacting with syntaxin-1 and accompanies it to the plasma membrane (37, 59).

Clinical studies found that the GLP-1 secretory rhythm of obese individuals is lost, providing support to the idea that obesogenic feeding also affects diurnal GLP-1 secretion (60). In addition, GLP-1 secretory rhythms have also been modified in morbidly obese T2D patients in comparison to a group having standard tolerance for glucose (60). Exposing rat’s GLUTag L cells to palmitate as a saturated fatty acid, which was a key constituent of the obesogenic Western diet, impairs circadian glucagon-like peptide-1 secretion (61).

The rhythm in GLP-1 release was paralleled by identical peak and trough insulin responses by β cells (52). Continuous GLP-1 receptor activation also increases insulin synthesis and β-cell proliferation and neogenesis (62). Exposure of human islets to melatonin for 12 h increases the sensitivity of the β cell to the stimulatory effects of GLP-1 (63). Thus, it appears likely that the human β cell shows a circadian pattern in its response to GLP-1 (63).

In chronotherapy, medicine is administrated at a specific time of the day, maximizing therapeutic response(s) and minimizing negative effects by benefiting from the circadian rhythms in physiology (**Table 2**). The dual GLP-1R/GIPR agonist DA-JC1 has been found to exert a stronger hypoglycemic effect than a GLP-1R agonist alone and could effectively improve the decline of learning and memory and circadian rhythm disorders induced by Aβ31-35 in mice (76).

**Table 2:** Biological functions of GLP-1 in target organs.

Organ	Effects	Reference
Pancreas	Insulin synthesis and secretion, B-cell proliferation	(64, 65)
GIT	Slowing gastric motility and emptying	(66)
Cardiovascular system	Improves endothelial function	(67, 68)
Organ inflammation	Anti-inflammatory benefits	(69)
Kidney	Elevates the flow of the renal plasma and filtration of the glomerulus	(70, 71)
Bone	Restoration of loss of bone mineral density	(72)
Liver	Induces the liver for gluconeogenesis, glycolysis and glycogen storage	(73)
Gonads and fertility	Increases the development of ovarian follicles, reduces testosterone concentrations	(74)
Brain	Neuroprotection, hypothalamic control of appetite	(75)

## Oxyntomodulin

A circadian pattern of release is shown by OXM, which is co-produced with GLP-1 in the L cell in the intestine, where the peak release occurs between the dark/active period (77). OXM functions through the receptors of both hormones GLP-1 and glucagon receptors by decreasing body weight and enhancing glucose metabolism (77, 78). The homeostatic functions of OXM on the body’s energy are exerted by enhancing the use of energy through the glucagon receptor and reducing energy intake, most likely through hypothalamic and area postrema activation by GLP-1R signaling (79, 80). Acute OXM infusion improves glucose tolerance in T2DM patients making dual agonists of the glucagon receptors and GLP-1R new promising treatments for diabetes and obesity with the potential for weight loss and glucose lowering superior to that of GLP-1R agonists (81, 82).

## Peptide YY

Peptide YY (PYY) is another anorexigenic hormone that is released by the L cells. In humans, the peak levels occur during the day (83). PYY can be characterized as having a meal-driven diurnal rhythm, as illustrated by significant correlations between PYY and meal timing as well as caloric load of a meal eliciting postprandial responses and contributing to the 24-h profile (84). Fasting as well as postprandial PYY concentrations has been shown to be depressed in obese and elevated in energy-deficient women (84). Interestingly, Guo et al. (85) found a negative correlation between fasting PYY and resting metabolic rate in humans. It is imperative to perform additional studies to explain the function of the circadian release of PYY to balance the diurnal rhythm of energy production in the body. Digestion is slowed down by PYY, which functions like an “ileal brake” in order to induce a better rate of absorbing nutrients (86). The highest concentrations are reached by PYY up to 2 h postprandially, which helps in meal cessation (87). Therefore, PYY is recognized as a satiety hormone.

## Neurotensin

Neurotensin is a hormone produced by N cells found in the distal part of the small intestine and facilitates fat ingestion and absorption (88), which in turn stimulates neurotensin release in animals and humans (89). It has been found that neurotensin also exhibits circadian rhythms. There was a significant 24-h rhythm in the levels of neurotensin in groups of rats maintained under constant illumination or a 12:12 light:dark cycle and fasted for either 24 h or provided food *ad libitum*. The levels of neurotensin were the highest during the early morning (04:00–08:00 h) and the lowest during the afternoon (12:00–16:00 h) in rat small intestines (90). There is a close relationship between neurotensin, intestinal GLP-1, and PYY, and the three peptide-regulating hormones are expressed, preserved, and secreted at the same time to manage peripheral and central metabolic targets (91). The essential processes of digestion and nutrient absorption that take place in the small intestine are dependent on the constant proliferation and differentiation of intestinal epithelial cells (84). This continuous self-renewal process is mediated by intestinal stem cells, which integrate dietary signals to maintain intestinal homeostasis. Both neurotensin and GLP-2 induce mucosal growth and proliferation and promote intestinal repair after inflammatory damage (92). At the molecular level, the GLP-1 receptor and the neurotensin receptor 1, which is dominating in the periphery, are coupled to Gs and Gq/11, respectively, which are signaling pathways that are well established to act synergistically (93).

## Enteroendocrine hormones and pregnancy

**Figure 2:**
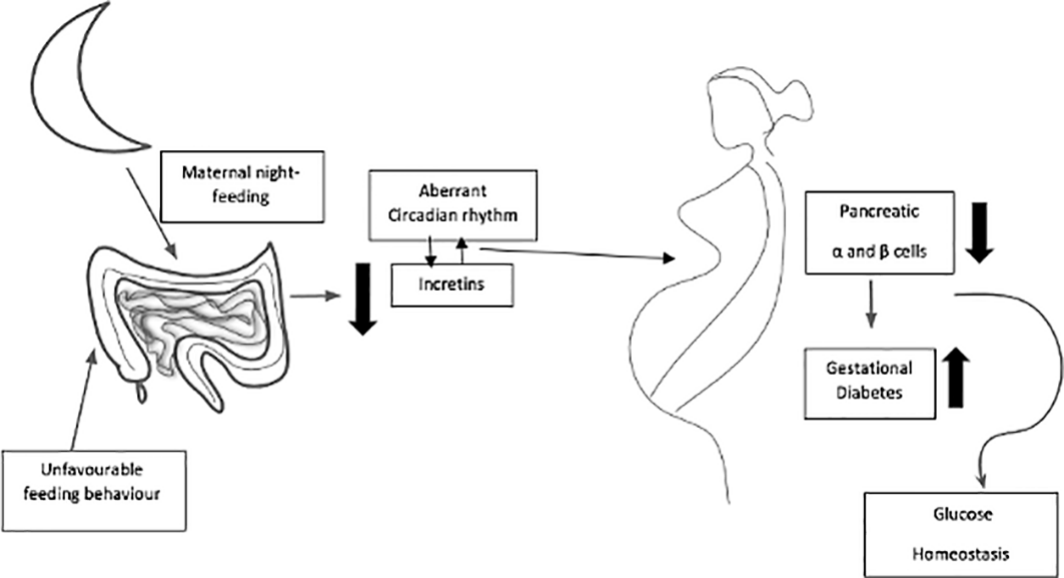
Maternal dark-period energy intake at night-time can together affect circadian rhythm of incretins, glucose tolerance and function of β-cells in pregnancy, and expose to high possibility of acquiring gestational diabetes mellitus.

Normal pregnancy requires a functional circadian system (94–97), and circadian disruption (**Figure 2**) can perturb pregnancy outcomes (98, 99). At approximately day 7 of pregnancy, mice shift their activity to a time that is 4 h earlier than for non-pregnant animals. In women, the timing of sleep onset is earlier during the first 27 weeks of gestation (100). In women, the onset of labor most commonly occurs between midnight and 4:00 a.m (101). Similarly, rodents are most likely to deliver pups at around dawn (101, 102). In pregnant rats, the ablation of SCN abolishes the preferential clustering of pup deliveries around dawn (103). Finally, women who work at night or on rotating shifts are 60% more likely to miscarry or deliver preterm than women who work day shifts (104, 105).

The enteroendocrine hormones PYY, GLP-1, and GLP-2 are co-secreted from the gut L cells (91) and have been linked with gut growth (16, 106) and increased capacity for nutrient absorption (98). Circadian rhythms of enteroendocrine hormones could be an additional mechanism to support pregnancy, increasing the surface area and gut capacity to process nutrients from more food. On day 4, pregnant rats had the largest ascending colon circumferences, with higher values in the duodenum and descending colon than in controls (107). These data may reflect the earliest pregnancy adaptations to rapidly increasing capacity, especially of the cecum to hold more food and facilitate microbe diversity changes (108). Hormonal (PYY/GLP-1) stimulation is needed to initiate and maintain gut growth upon extensive organ remodeling (99, 109). PYY and GLP-1 are co-secreted, with GLP-2 (91) having been previously linked with gut growth in adult female mice and rats (110, 111). PYY and GLP-2 cause substantial intestinal hypertrophy, which highlights the role of L cells and their secretory products after gut surgeries for body mass reduction, as these techniques lead to rapidly increased concentrations of appetite-regulating hormones (112) and may stimulate intestinal growth in attempts to regenerate the remaining gut tissues (112).

In the initial days of gestation, maternal metabolism is an anabolism process that manages the higher requirement of energy for fetal and placental growth in the later stages of gestation. Multiple biomolecules including glucose, fatty acids, ketone bodies, and hormones collectively contribute toward these metabolic adaptations. This is because of elevated concentrations of circulating blood glucose to meet the increasing requirements of the growth and metabolism of both placental and fetus development and metabolism (113). Changes in maternal release and sensitivity of insulin that take place during different stages of pregnancy are important for regulating these changes in energy utilization (113). It has been determined that there is a greater prevalence of insulin resistance and maternal obesity during pregnancy (114). Research carried out in North America and Scandinavia (115) shows the adverse effects of these alterations on carbohydrate metabolism, fetal development *in utero*, and neonatal health subsequently. Excess maternal weight gain in pregnancy contributes to a glycemic environment and insulin resistance that affects fetal growth (95). Maternal GLP-1 might be involved in mechanisms that compensate for the pregnancy-related increase in glycemia and insulin resistance, suggesting a role of this peptide in maternal metabolism and weight and fetal growth (116). Lowered levels of GIP and GLP-1 may play an important role in the abnormality of glucose regulation following pregnancy. Serum GIP and GLP-1 levels were inversely associated with gestational diabetes mellitus (GDM), and participants with lower levels of GIP and GLP-1 had a seven-fold higher risk of developing GDM compared with the higher levels, suggesting that there is an independent, inverse association between fasting incretins and higher risk of GDM (114).

It has been demonstrated in experimental studies on mice that the quantity of maternal intestinal GLP-1-secreting L cells in the maternal intestine increases during pregnancy (117). Pregnancy is associated with the physiological and reversible expansion of β-cell mass (117). Incretin hormones inhibit β-cell apoptosis and stimulate proliferation, resulting in the development of β-cell mass (54). Islet adaptations to pregnancy were explored in C57BL6/J mice lacking functional receptors for GLP-1 and gastric inhibitory polypeptide (GIP). The data collected indicated that GLP-1 but not GIP is a key mediator of β-cell mass expansion and related adaptations in pregnancy, triggered in part by the generation of intra-islet GLP-1 (117).

Alterations in circadian rhythms are brought about by maternal adjustment (117–121), with significant modifications in the expression pattern of diurnal clock genes (122). The variations in mother gene expressions of peripheral clocks throughout gestation specifically bring about downstream changes in the diurnal expression of particular metabolism genes, such as the glucoregulatory genes *Pck1*, *Gk*, and *G6Pase*, to ensure a healthy pregnancy (122). This shows that when there are interruptions in circadian rhythms during pregnancy, women may be at risk of acquiring metabolic diseases and experiencing negative effects of gestation. It was determined that night-shift pregnant employees face more risks of prematurity, miscarriage, hypertensive disorders, and low birth weights (123). These results are not only relevant to night-shift pregnant employees but are likewise applicable to pregnant women population who consume high-energy intakes in the evening or in their night shift, with possible chronodisruption. The human body is evolved for rest at night. Observations in pregnant women are in agreement with evidence in men, indicating that food timing and the amount of dietary carbohydrates could affect glucose metabolism (123–125). A dietary carbohydrate constitution (for example, highly *vs*. poorly digestible carbohydrates) has a significant impact on the clock mediating glucose homeostasis (125).

Maternal dark-period energy intake and the volume and form of intaking carbohydrates at night can together affect glucose tolerance and the function of β cells in pregnancy and exposure to the high possibility of acquiring gestational diabetes mellitus. Here, the triggered or natural circadian release of enteroendocrine hormones may play a role as an insulinotropic. A significant research topic that is of interest to scientists and nutritionists/dietitians focuses on nutritional programs to attain optimal results in pregnancy. It seems that the timing of intaking energy and diet constituents could be used to mitigate metabolic disturbances that may happen throughout pregnancy, in addition to the safety impact on gestation results. It can be concluded from the few studies presented that intaking a high-energy source at nighttime and its diet component may contribute to impaired energy and glucose metabolism and reproductive hormone disruption during pregnancy (126, 127). In pregnant animals, it was found that disturbed intake timing, or feeding (during the day period by nocturnal rodents), gave rise to an irregular circadian rhythm in the mother and fetus (127) and was capable of altering the microbiota profiles to give rise to metabolic disorders (128). However, studies that translate these mechanisms to pregnant women have not yet been carried out.

The importance of the circadian clock in maintaining human health is now widely acknowledged. Circadian rhythms can act as therapeutical targets, therefore managing the factors that influence the clocks. Some of the potentials as chronotherapeutic agents are flavonoids. Nobiletin, a polymethoxy flavone obtained from dried citrus peel, known as *Citri Reticulatae Pericarpium* fruit, was identified as a particularly effective clock amplitude-enhancing small molecule and can directly affect the mammalian circadian system (128–130). Time-restricted feeding partially restores diurnal rhythms of the ileal microbiome (131). Interestingly, regular fasting periods may provide physiological benefits such as improved circadian rhythmicity and modulation of the gut microbiota (127).

## Conclusion

It is demonstrated in the current review that under physiological conditions, the enteroendocrine hormones GLP-1, neurotensin, GIP, PYY, and OXM play a role in regulating vital metabolic functions through circadian secretion rhythms. A synergistic effect may be indicated by their secretion, co-expression, and action on metabolism when administered in pharmacological doses. A vital theme of research for scientists is considering diet programming as a new strategic policy to achieve optimal pregnancy results. Changes in circadian patterns are brought about by maternal modification during gestation, with significant differences in circadian clock gene expression. Disturbances in metabolism in pregnancy that may lead to negative results could be addressed by possible novel methods of programming the timing of energy intake and diet components.

## Author contributions

All authors contributed to the article and approved the submitted version.
